# Hybrid Resorbable 3D-Printed Mesh/Electrospun Nanofibrous Drug/Biomolecule-Eluting Mats for Alveolar Ridge Preservation

**DOI:** 10.3390/polym15163445

**Published:** 2023-08-18

**Authors:** Shuen-Yeo Chen, Fu-Ying Lee, Ren-Chin Wu, Chien-En Chao, Chia-Jung Lu, Shih-Jung Liu

**Affiliations:** 1 Department of Periodontics, Division of Dentistry, Chang Gung Memorial Hospital at Linkou, Taoyuan 33305, Taiwan; jameschenhy21@gmail.com (S.-Y.C.); fuying20@hotmail.com (F.-Y.L.); 2 Department of Pathology, Chang Gung Memorial Hospital, Chang Gung University, Taoyuan 33305, Taiwan; renchin.wu@gmail.com; 3 Department of Mechanical Engineering, Chang Gung University, Taoyuan 33302, Taiwanhappy2231017@mail.cgu.edu.tw (C.-J.L.); 4 Department of Orthopedic Surgery, Bone and Joint Research Center, Chang Gung Memorial Hospital at Linkou, Taoyuan 33305, Taiwan

**Keywords:** alveolar ridge preservation, drug-eluting nanofibrous mats, 3D printing, coaxial electrospinning

## Abstract

In this research study, we developed hybrid resorbable three-dimensional (3D)-printed mesh/electrospun nanofibrous biomolecule-eluting mats for alveolar ridge preservation. The fabrication process involved the use of 3D printing and coaxial electrospinning technologies. Specifically, we utilized a lab-developed solution-extrusion 3D printer to fabricate polycaprolactone (PCL) meshes. Then, bi-layered poly(lactic-co-glycolic acid) (PLGA) nanofibrous membranes, which embedded ibuprofen and epidermal growth factor (EGF), were prepared utilizing electrospinning and coaxial electrospinning techniques, respectively. To ensure the quality of the produced mesh and spun nanofibers, we carried out a characterization process. Furthermore, we estimated the in vitro and in vivo release characteristics of ibuprofen and EGF, respectively, using high-performance liquid chromatography and enzyme-linked immunosorbent assays. In addition, we assessed the effectiveness of hybrid nanofibrous mats for preserving the alveolar ridge by adopting an animal model and conducting a histology examination. The study findings demonstrate that the nanofibrous mats provided a continuous discharge of ibuprofen and EGF for more than four weeks. Moreover, the animal test carried out in vivo showed that animals implanted with this combination of mesh and drug-eluting mats displayed considerably greater mobility than those without mats. The histological analysis revealed no unfavorable impacts from the drug-eluting mats. Our study demonstrated the successful fabrication of resorbable drug-eluting nanofibrous mats for alveolar ridge preservation by utilizing both 3D printing and coaxial electrospinning technologies.

## 1. Introduction

In our routine dental practice, alveolar ridge preservation (ARP) has been proposed as a post-extraction treatment to minimize the physiologic changes of the alveolar ridge surrounding the extraction socket, reduce the need for auxiliary ridge augmentation procedures, and enhance therapeutic predictability [1,2,3]. This involves filling the extraction socket with a range of graft materials, such as autografts, allografts, xenografts, or synthetic grafts from a host or donor, and typically covering the tooth immediately after extraction [4,5]. Previous research has demonstrated that unaided alveolar bone healing may lead to an average of 29–63% horizontal bone loss and 11–22% vertical bone loss at a 6-month follow-up [6,7]. However, a consensus report from a workshop of the European Federation of Periodontology (EFP) in 2019 showed that compared to unassisted extraction sockets, ARP might prevent 1.5–2.4 mm of horizontal, 1–2.5 mm of vertical mid-buccal, and 0.8–1.5 mm of mid-lingual vertical bone resorption [8].

The evolution of modern technology has stimulated a keen interest in determining the most suitable materials and techniques for ARP [9]. However, there is currently no consensus on case selection, clinical technique, or material choice [8,10]. Ideal grafting material for ARP must own suitable biomechanical strengths to offer reinforcement or barrier, transport the proper drug/biomolecule levels to the targeted area for pain relief and healing enhancement, and be resorbable and biocompatible to prevent adverse tissue responses during the biodegradation process. Hydrogels have been developed as scaffold materials in the tissue engineering area for repairing dental pulp and periodontal damages [11,12,13]. However, hydrogels are often soft and have low mechanical strength. This can be a disadvantage when used in dental restorations or devices that require higher durability and load-bearing capacity. Hydrogels may not adhere well to natural tooth structures or other dental materials, which is crucial for ensuring the stability and longevity of dental restorations. Furthermore, hydrogels have a tendency to absorb water and swell. In the oral environment, exposure to saliva and other fluids could lead to unpredictable changes in the hydrogel’s size and shape, affecting its performance and stability.

We have engineered a novel approach for alveolar ridge preservation by the integration of three-dimensional (3D)-printing and electrospinning/coaxial electrospinning processes. Our solution involves the production of hybrid resorbable membranes that release biomolecules, utilizing polycaprolactone (PCL) meshes fabricated via a lab-developed solution extrusion 3D printer and bi-layered poly(lactic-co-glycolic acid) (PLGA) nanofibrous membranes that are loaded with ibuprofen and epidermal growth factor (EGF) through electrospinning and co-axial electrospinning techniques, respectively.

Three-dimensional printing is widely recognized as an innovative technology that has the potential to address complex biomedical challenges [14]. The versatility of this technique facilitates innovation and enables significant design flexibility. With its tool-free process and relatively low investment requirements, 3D printing offers ample opportunities for personalized diagnostics and treatment [15]. Electrospinning is an efficient method for producing nanofibrous mats, characterized by a nanofibrous network with a three-dimensional structure and a significant surface area that can simulate native extracellular matrices [16,17]. Moreover, the potential for using nanofibers in tissue engineering and drug delivery applications is emphasized by the ability to tailor them to achieve sustained release of biomolecules and drugs. Coaxial electrospinning, conversely, is a fast and efficient method for manufacturing nanofibrous membranes with a sheath-core structure [18]. One significant advantage of this method is the ability to encapsulate functional macromolecules such as proteins and peptides in a single step. Since the biomolecules are protected within the core, the risk of exposure to organic solvents during spinning is minimized, thereby preserving their biological activity [19].

With a melting temperature of 59–64 °C and a glass transition temperature of −60 °C, PCL is a semi-crystalline polymer that is widely used in the production of resorbable sutures, regenerative therapy scaffolds, and drug delivery systems because of its biocompatibility and non-toxic properties. The aliphatic ester linkage in PCL is subject to hydrolysis under physiological conditions, allowing for its absorption either through microorganisms or over an extended period of more than two years [20]. PLGA is a degradable biopolymer that is extensively adopted in drug delivery because of its outstanding biodegradability, biocompatibility, and capacity to enhance the delivery of proteins and biomolecules [21].

Ibuprofen belongs to a class of nonsteroidal anti-inflammatory drugs (NSAIDs) commonly used to alleviate pain associated with a range of conditions, including but not limited to headaches, dental pain, menstrual cramps, muscle aches, and arthritis. In the field of dentistry, ibuprofen has been widely prescribed for post-operation analgesics [22,23]. Its mechanism of action involves peripheral inhibition of cyclooxygenases, resulting in subsequent prostaglandin synthetase inhibition [24]. Epidermal growth factor (EGF) is a protein that activates cell growth and differentiation by binding to its receptor. This biomolecule consists of a single polypeptide chain composed of 53 amino acid residues, and it plays a crucial role in regulating cell proliferation and wound healing [25,26]. The effects of EGF on target cells are mediated by its binding to the plasma membrane-localized EGF receptor.

Following fabrication, the printed mesh and spun nanofibers were subjected to comprehensive characterization of their properties. The in vitro and in vivo release kinetics of ibuprofen and EGF incorporated in the nanofibers were determined using high-performance liquid chromatography (HPLC) and enzyme-linked immunosorbent assays (ELISA), respectively. Furthermore, the efficacy of the hybrid nanofibrous mats in preserving the alveolar ridge was assessed on a rat model, and the activities of the implanted rats were evaluated. Additionally, a histological assay was conducted to further elucidate the results.

## 2. Materials and Methods

### 2.1. Materials

In this study, PCL (molecular weight: 80 kDa) was used for the mesh material, and dichloromethane (DCM) was adopted as the solvent, both of which were purchased from Sigma-Aldrich (Saint Louis, MO, USA). The nanofibers comprised PLGA with a molecular weight of 33,000 Da and a 50:50 ratio of lactic acid to glycolic acid, using hexafluoroisopropanol (HFIP) from Sigma-Aldrich as the solvent. Recombinant enhanced green fluorescent protein (reGFP) was acquired from Shanghai PrimeGene Bio-Tech, Shanghai, China. Ibuprofen and epidermal growth factor (EGF) were loaded into the nanofibers and were also obtained from Sigma-Aldrich.

### 2.2. D-Printing of PCL Meshes

**Figure 1:**
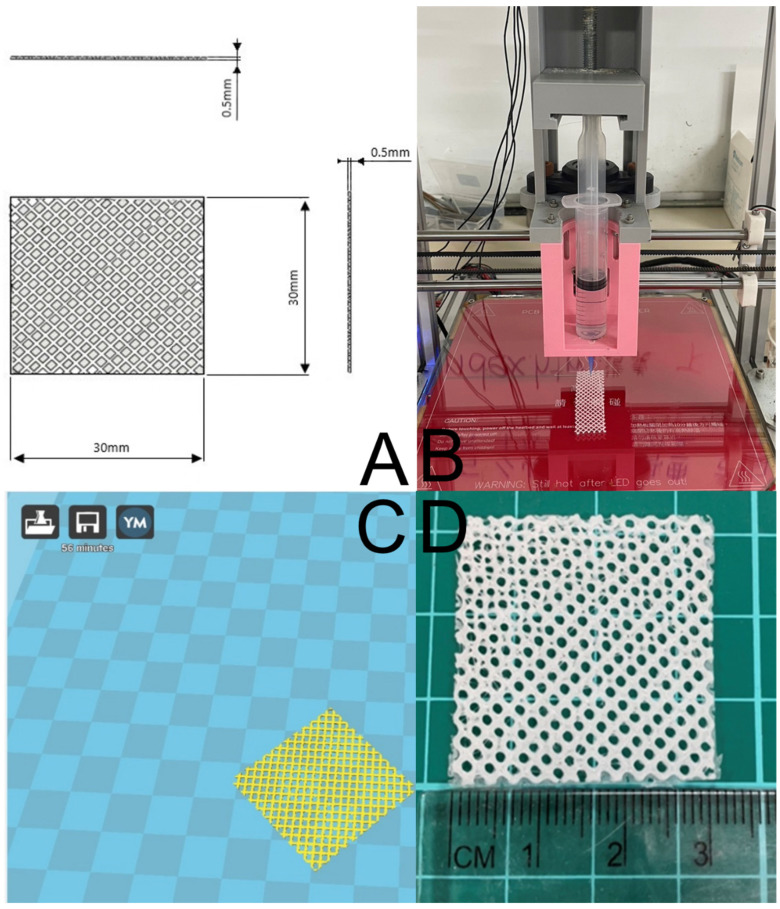
(**A**) Layout and dimensions of PCL mesh, (**B**) the solution-extrusion 3D printer, (**C**) the Ultimaker Cura interface used to manage the printing process, and (**D**) the printed PCL mesh.

Figure 1A shows the layout and dimensions of the PCL mesh. The present study involved the fabrication of degradable meshes using a laboratory-developed solution-extrusion 3D printer (Figure 1B) [27]. To achieve this, PCL (2500 mg) was combined with DCM (3.5 mL) using a magnetic stirrer for a period of 2 h. Next, the extrusion setup of the 3D printer was loaded with the PCL-DCM mixture, which included a nozzle/syringe to print the parts. The printing process employed a Cura code (Figure 1C) to direct the motor and nozzle, allowing the PCL mixture to be extruded onto a collection sheet. As the solvent evaporated, PCL meshes were then acquired (Figure 1D).

### 2.3. Preparation Ibuprofen and EGF Incorporated Nanofibers

Drug and biomolecule-loaded bi-layered nanofibrous membranes were manufactured by creating a regular layer and a sheath-core structural layer. Several preliminary trials were conducted to determine the appropriate polymer and drug compositions necessary for the successful production of the nanofibrous membranes. The ordinary nanofiber layer was produced by mixing PLGA (1120 mg) and ibuprofen (280 mg) with 5 mL of HFIP and electrospinning them using a laboratory-developed spinning apparatus at a rate of 0.7 mL/h.

Once the regular layer was electrospun, the sheath-core structural layer was created. PLGA (1400 mg) was blended with 5 mL of HFIP to serve as the material for the sheath layer, while the solution at the core was composed of 1 mL of EGF (20 μg) blended with 1 mL of phosphate-buffered saline (PBS). To spin the nanofibers, a dedicated coaxial spinning device that simultaneously delivers two solutions was used [28]. The solutions were then electrospun and delivered to the collection sheet at volumetric flow speeds of 0.9 mL/h for the shell PLGA and 0.3 mL/h for the core EGF utilizing two different pumps. The spinning trials were conducted at room temperature, with the voltage set at 17 kV and the travel distance for the spun solution to the collection plate set at 15 cm.

The outcome of the experiment yielded hybrid bi-layered nanofibrous membranes, with each layer having a thickness of about 0.1 mm and a total thickness of approximately 0.2 mm. To remove the solvent, all electrospun nanofibers were kept in a vacuum oven at 40 °C for three days and then stored at 4 °C till required.

### 2.4. Mechanical Property

The tensile strength analysis of fabricated PCL meshes was conducted using a Lloyd tensile testing device manufactured by Ametek in the United States, with a load cell of 2500 N. The ultimate strengths were recorded while maintaining the extensional rate at 6 cm/min. Additionally, the ultimate load and deformation were acquired to evaluate the tensile properties of both pure PLGA and biomolecule-loaded PLGA using an elongational rate of 60 mm/min.

### 2.5. Microscopic Observations

The morphological structure of the electrospun nanofibers was evaluated by means of scanning electron microscopy (SEM). To assess the nanofiber diameter distribution, 100 fibers were randomly selected (*N* = 3), and an ImageJ code (National Institutes of Health, Bethesda, MD, USA) was used.

A JEOL Model JEM-2000EXII transmission electron microscope (TEM) from Tokyo, Japan, was used to confirm the sheath/core structure of coaxial electrospun nanofibers. To verify the presence of active biomolecules, PLGA was used as the sheath material and reGFP at the core in the electrospinning process. The resulting nanofibers were observed under a Leica TS SP8X laser scanning confocal microscope (LSCM) from Tokyo, Japan, at a wavelength of 487 nm.

### 2.6. Fourier Transform Infrared Spectroscopy

To acquire the spectra of ibuprofen-loaded nanofibers, Fourier transform infrared (FTIR) spectroscopy was conducted using a Bruker Tensor 27spectrometer (Billerica, MA, USA) with an absorption mode and 4 cm^−1^ resolution. A total of 32 scans were performed on the nanofibrous specimens, which were pressed into KBr discs and observed within the span of 400~4000 cm^−1^.

### 2.7. Differential Scanning Calorimetry

The thermal characteristics of pristine PLGA and PLGA nanofibers embedded with ibuprofen were evaluated through differential scanning calorimetry (DSC), employing a DSC25 instrument manufactured by TA Instruments in New Castle, DE, USA. The temperature range employed was 30 to 250 °C, with a heating speed of 10 °C/min applied to the specimens.

### 2.8. Hydrophilicity

To evaluate the hydrophilicity of the PLGA nanofibers, water contact angles of both pristine PLGA and drug and biomolecule-incorporated PLGA nanofibrous membranes were determined. A controlled amount of distilled water was applied to the surface of a 10 mm^2^ section of the nanofiber samples and subsequently measured (*N* = 3).

### 2.9. In Vitro Discharge of Drug and Biomolecule

To investigate the release kinetics of ibuprofen from drug-eluting nanofibers, in vitro elution analysis was performed. 200 mg nanofibrous sample with a size of approximately 2 cm × 3 cm was put in assay tubes (*N* = 3) holding 1 mL of PBS (Sigma-Aldrich) and kept at 37 °C for 24 h. The solution in the tubes was substituted by fresh PBS (1 mL) every 24 h for a period of 30 days.

The Hitachi L-2200R HPLC system (Tokyo, Japan) was used to evaluate the drug concentrations in the gathered media (*N* = 3). The assay employed a SunFire^®^ C18 (25 cm × 4.6 mm × 5 μm), and the mobile phase for the ibuprofen was a mixture of acetonitrile: (1000 mL deionized water + 2 mL triethylamine, with the pH value adjusted to 3.2 by phosphoric acid) (50:50, *v*/*v*). The assay wavelength was 280 nm, and the flow speed was 1.5 mL/min, with a retention time of 7 min.

Meanwhile, ELISA was used to measure EGF levels. 100 µL of eluents were put into the corresponding microtiter plate wells along with a biotin-conjugated antibody and incubated at 37 °C for 90 min. Each well of the microplate was treated with avidin that had been linked to horseradish peroxidase (HRP) and then allowed to incubate for a period of 30 min. The color change was measured spectrophotometrically at 450 nm after the addition of a sulfuric acid solution. The EGF level in the eluents was determined by comparing the optical density of the samples to a standard curve. The experiments were conducted in triplicate (*N* = 3).

### 2.10. Animal Studies

#### 2.10.1. Animal-Related Procedures

Sprague–Dawley rats with an average weight of approximately 600 g were procured and nurtured in compliance with the guidelines set forth by the Department of Health and Welfare, Taiwan. The Institutional Animal Care and Use Committee of Chang Gung University (CGU110-105) approved the animal-related protocols.

**Figure 2:**
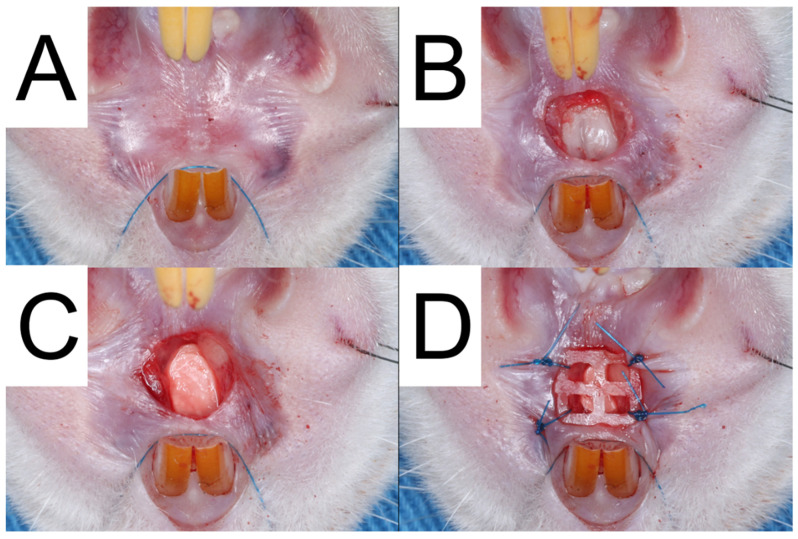
The surgical procedure performed on Group A was documented through a series of photographs. The first photograph (**A**) displays the pre-operative condition of the palate. The second photograph (**B**) exhibits the removal of gingival tissue and bone to mimic extraction sockets. The third photograph (**C**) displays the implantation of bilayer drug-embedded nanofibers into the socket. Finally, the fourth photograph (**D**) illustrates the use of a 3D-printed PCL mesh to prevent implant loss.

To mimic an extraction socket at the upper palate, the rats were initially anesthetized using Tiletamine + Zolazepam (0.06 mL per 100 g body weight, Zoletil 50) and xylazine hydrochloride (0.05 mL per 100 g body weight, Rompun) through Intraperitoneal injection. Figure 2 illustrates the procedure in which the rat’s head was immobilized using sutures, and the subsequent steps were carried out.

Initially, a 3.0 mm-diameter biopsy punch from Integra^®^ Miltex^®^, Princeton, NJ, USA, and a #15c stainless steel scalpel were utilized to delicately remove the full-thickness gingival tissue using a sharp tissue elevator. Subsequently, a 3.0 mm round carbide bur (30,000 Revolutions Per Minute) was used to create a 3.0 mm diameter and 1.5 mm deep socket after raising the mucoperiosteal flap.

The rats were then randomly assigned to three groups: In Group A (*N* = 3), bilayer drug-embedded nanofibers were implanted in the alveolar socket. Subsequently, the 3D-printed PCL mesh was fixed over the socket wound with 6-0 polypropylene sutures (Prolene; Ethicon, Raritan, NJ, USA). Conversely, rats in Group B (*N* = 3) received nanofibrous mats without embedded drugs, and the 3D-printed PCL mesh was immobilized as in Group A. In Group C (control group), the alveolar sockets of the rats were created similarly to the test groups, and natural secondary wound healing was performed without assistance. Another three animals that did not receive any surgery were employed as the healthy group.

Local tissue drug concentrations in rats from Group A were collected on postoperative days 1, 3, 7, and 14 (*N* = 3 for each day). A #30 standardized sterile paper point (manufactured by DiaDent in the Republic of Korea) was used to extract tissue fluid from the palatal wound. The paper point was left in place for 30 s during each process to ensure adequate samples eluted with 0.1 mL of PBS immediately after being collected and stored at a temperature of −20 °C until analysis. The HPLC analysis was conducted to measure the drug levels at each time interval, using the same steps as that of the in vitro study. In addition, systemic drug concentration was determined by drawing blood specimens using syringes from the rats’ tails on days 1, 3, 7, and 14 (*N* = 3 for each day).

#### 2.10.2. Post-Surgical Evaluation

**Figure 3:**
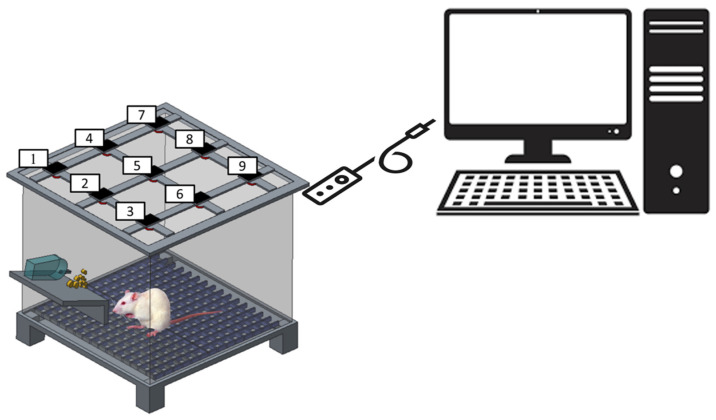
Schematic of the rat cage equipped with movement sensors (the numbers denote the locations of sensors).

The postoperative behavior of each rat within the study groups was evaluated utilizing an activity cage. Nine sensors were installed on top of the cage (Figure 3) and monitored for a duration of seven days. Each rat was individually placed inside the cage, and the sensors were triggered as the animal advanced from one district to another within the cage.

#### 2.10.3. Wound Healing and Histology Analysis

The clinical wound healing process was evaluated using standardized intraoral digital photographs taken at an angle of 90 degrees to the palatal surface across all groups on days 1, 3, 7, and 14. Healing analysis was conducted by utilizing Image J software version 1.44 to measure the wound area’s margins digitally. The area of the wound was measured at each designated time point. Histological specimens were obtained from rats belonging to Groups A and C. Standard biopsies were carried out on days 1, 3, 7, and 14 to assess tissue responses under microscopic examination. The harvested specimens were fixed in 10% neutral formalin, embedded in paraffin, and then cut into 4 μm sections. Hematoxylin and Eosin (H&E) staining were used to examine the epithelial and connective tissue characteristics.

### 2.11. Statistical Analysis

In order to assess variances between groups, paired *t*-tests were utilized to statistically analyze two-group comparisons. Statistical significance was established at *p*-values lower than 0.05.

## 3. Results

### 3.1. Characterization of Printed Meshes and Spun Membranes

**Figure 4:**
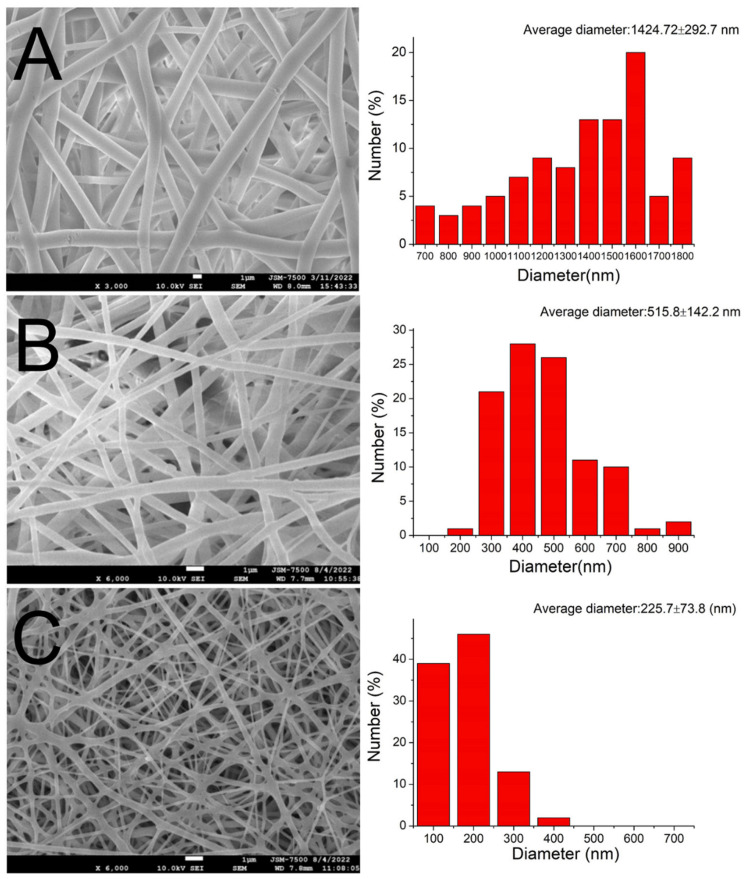
SEM images and fiber diameter distributions of (**A**) virgin PLGA, (**B**) ibuprofen-loaded PLGA, and (**C**) sheath-core PLGA nanofibers incorporated with EFG.

**Figure 5:**
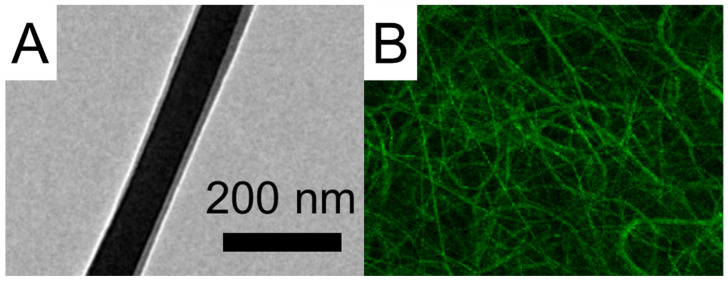
(**A**) TEM image and (**B**) LSCM image of sheath-core PLGA nanofibers incorporated with EFG.

Using 3D printing and electrospinning methods, we created a degradable hybrid mesh consisting of PCL and drug-eluting PLGA nanofibrous membranes intended for alveolar ridge preservation. The printed mesh exhibited good flexibility, which would facilitate its implantation during surgery. Additionally, the morphological structures of the spun nanofibers are depicted in Figure 4. The sizes of the virgin PLGA, ibuprofen-incorporated PLGA, and EFG-incorporated sheath-core PLGA nanofibers were 1425 ± 293, 516 ± 142, and 226 ± 74 nm, respectively. The TEM image in Figure 5A affirms the sheath-core structure of co-electrospun PLGA nanofibers. Furthermore, the presence of bioactive proteins in the co-electrospun nanofibers is further confirmed by the image depicted in Figure 5B.

The image clearly displays green strings of reGFP, indicating that the bioactivity of the biomolecules in the core of the sheath-core-structured nanofibers has been effectively preserved. The EFG-loaded nanofibers also exhibited smaller dimensions than the pristine nanofibers.

**Figure 6:**
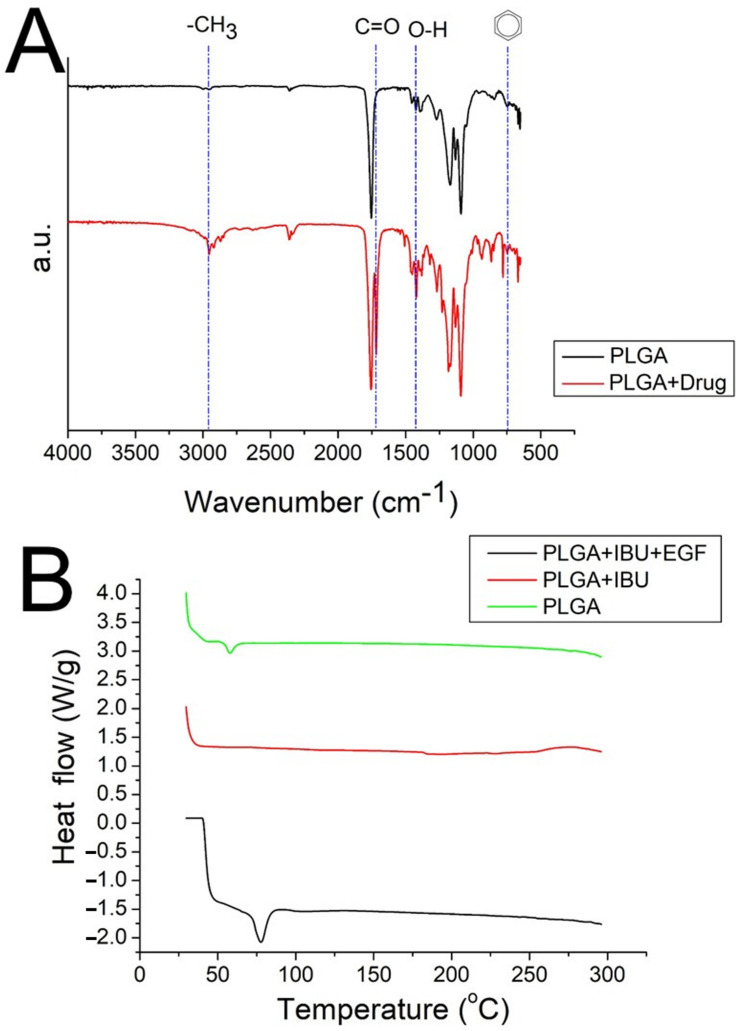
(**A**) FTIR spectra, (**B**) DSC thermogram of the virgin PLGA and drug-loaded PLGA nanofibers. (IBU: ibuprofen).

The results of the FTIR assay for pure PLGA nanofibers and ibuprofen-loaded PLGA nanofibers are shown in Figure 6A. The appearance of a new peak at 730 cm^−1^ indicated the presence of benzene from the added ibuprofen [29]. The vibration peaks corresponding to CH_3_, C=O, and O-H bonds were also enhanced owing to the addition of ibuprofen. Furthermore, Figure 6B depicts the DSC curves for both pristine PLGA and drug-incorporated PLGA nanofibers. The glass transition peak of PLGA at 56.7 °C diminished after mixing with ibuprofen [30]. Instead, the new exothermal peak at 78.9 °C, caused by EGF [31], was identified. These results collectively indicate that the biomolecules were successfully integrated into the PLGA nanofibers.

**Figure 7:**
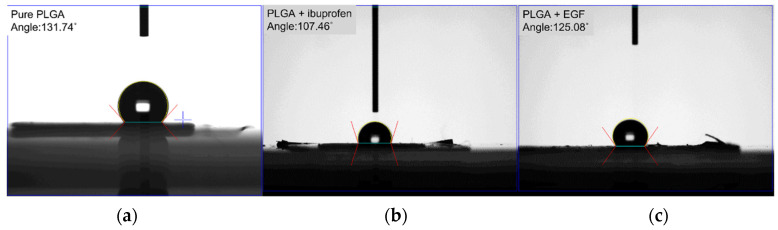
Wetting angles of (**a**) pure PLGA, (**b**) drug-loaded PLGA, (**c**) EGF-incorporated PLGA nanofibers.

The water contact angles of the pristine PLGA, ibuprofen-loaded PLGA, and EGF-incorporated PLGA nanofibers were estimated to be 131.74°, 107.5°, and 125.1°, respectively (Figure 7).

While the pristine PLGA and EGF-incorporated sheath-core PLGA nanofibers displayed hydrophobic features, the water-soluble ibuprofen in the drug-loaded PLGA mats enhanced the hydrophilicity of the spun nanofibers.

**Figure 8:**
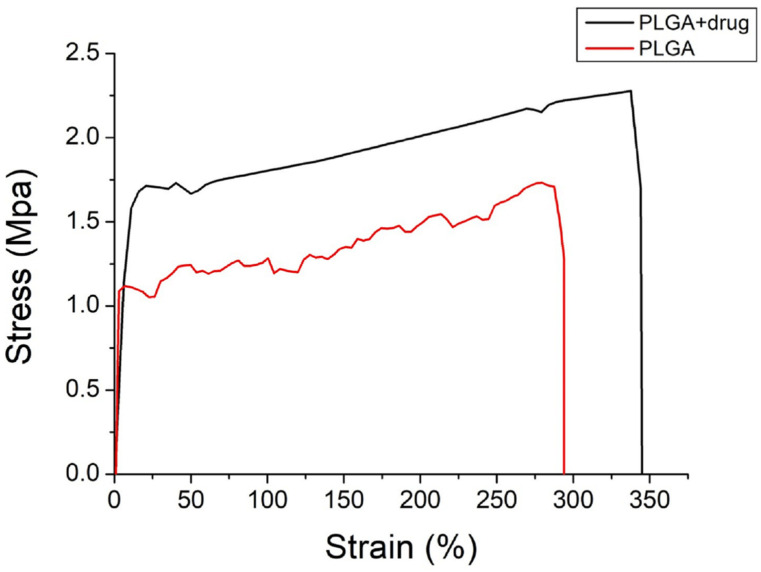
Tensile curves of electrospun nanofibers.

Figure 8 shows the tensile curve of pristine PLGA and ibuprofen/EGF-eluting PLGA nanofibers.

Despite the incorporation of drug and growth factor decreased the ultimate strength and elongation at the break of nanofibrous mats, the mats still exhibited excellent extensibility (with a strain of nearly 300%). This provides the advantage of coping with the extension/contraction during the healing process.

### 3.2. In Vitro/In Vivo Discharge

**Figure 9:**
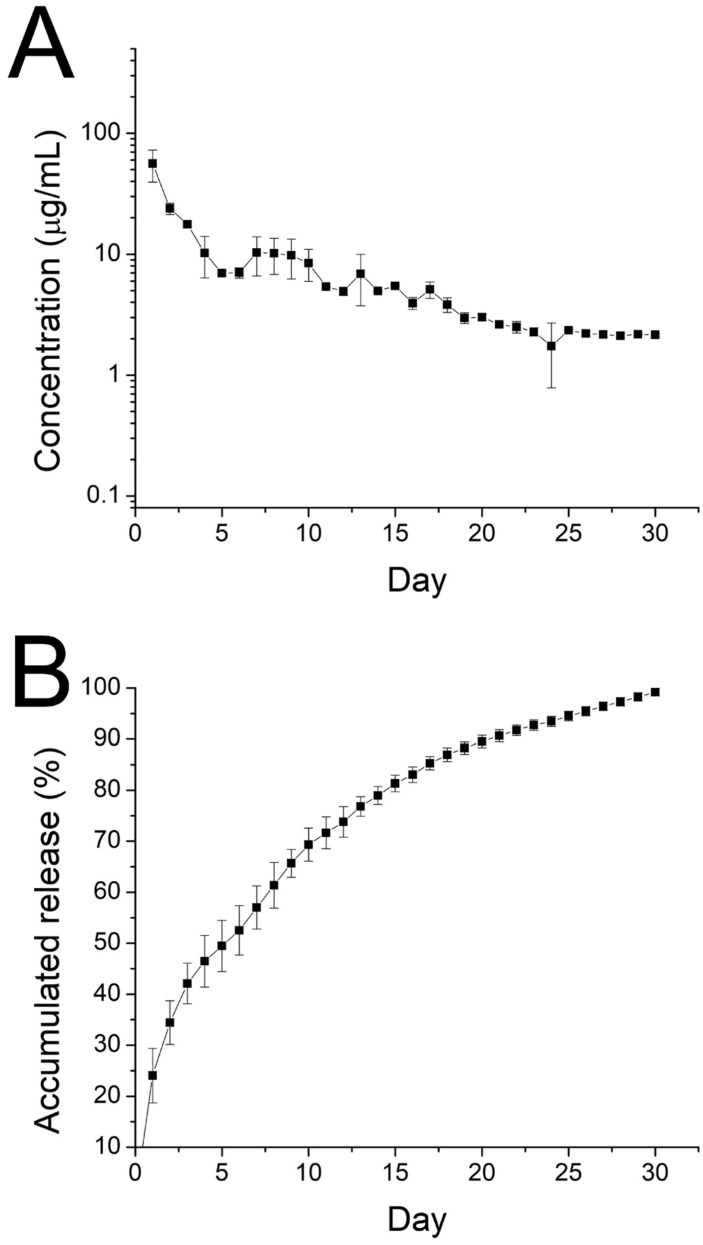
Curves displaying in vitro (**A**) daily and (**B**) cumulative release of ibuprofen from electrospun nanofibers.

The in vitro daily and cumulative profiles of ibuprofen released from the nanofibrous membranes are shown in Figure 9A,B, respectively.

**Figure 10:**
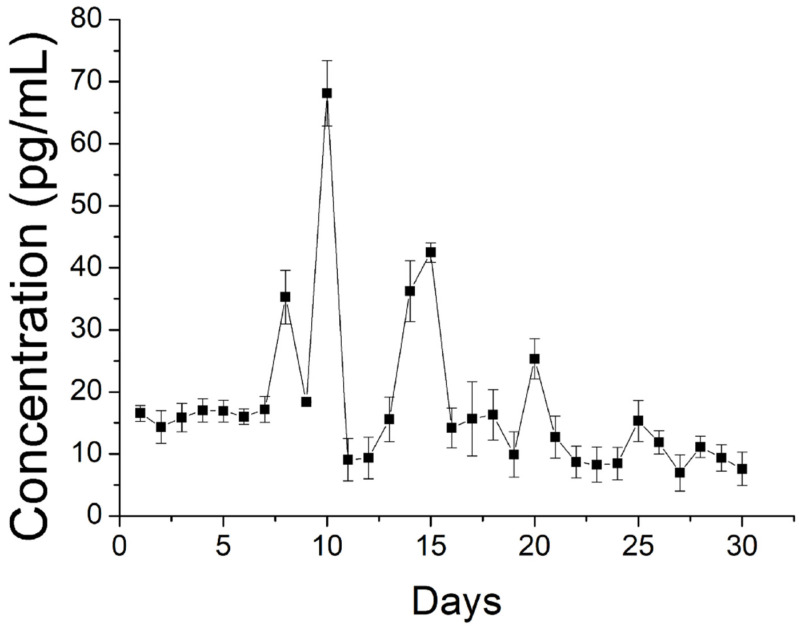
Release of EGF from spun nanofibers.

The drug exhibited a peak release at day 1, accompanied by a stable and gradual diminishing drug discharge for 30 days. Further, data in Figure 10 suggest that the sheath-core nanofibers could offer sustained discharge of EGF for 30 days.

Due to the shielding effect of the sheath PLGA material, no burst release was noted on day 1. Rather, a high release was found on days 9, 14, and 20, with gradually diminishing peaks.

**Figure 11:**
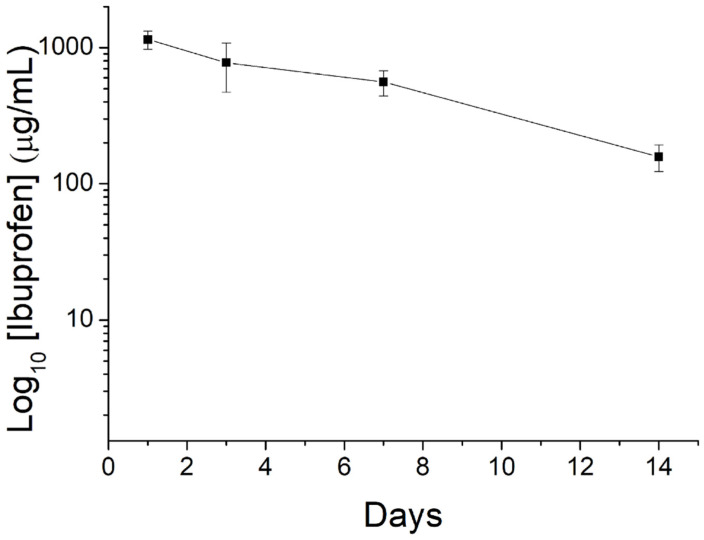
Curve displaying in vivo release of ibuprofen from the nanofibers.

Figure 11 displays the in vivo discharge characteristic of ibuprofen from the nanofibers.

The nanofibrous mats could offer extended drug release for over 14 days. Meanwhile, the drug level in blood was not detectable (lower than the lowest detectable range of 0.01 μg/mL of HPLC), demonstrating the advantage of drug delivery via degradable nanofibers that achieve high drug levels to the target site with minimized systemic side effects.

### 3.3. Animal Study

**Figure 12:**
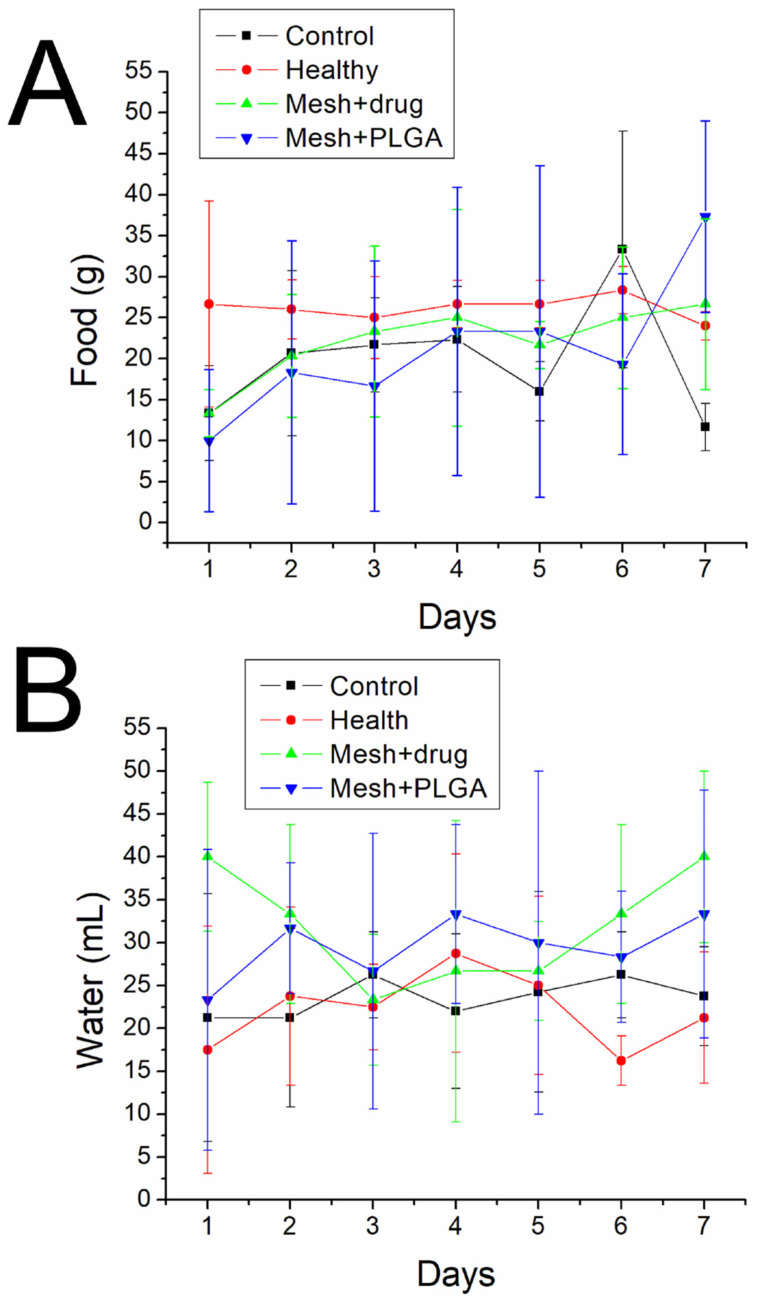
(**A**) Food and (**B**) water intakes of animals in various groups.

Figure 12 indicates that the animals in all groups consumed similar amounts of food and water.

**Figure 13:**
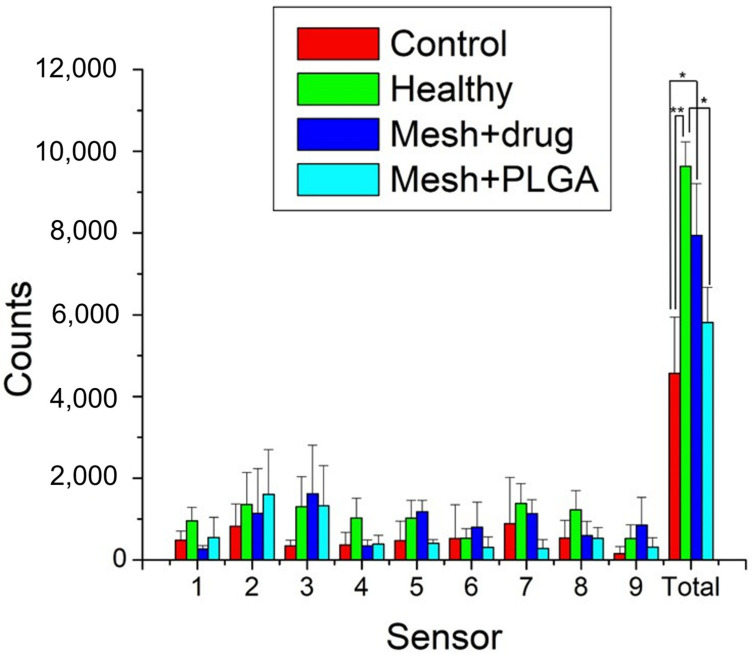
Rat activity counts over 7 days in the cage (**: *p* < 0.01, *: *p* < 0.05).

Additionally, the activity counts for each group over the 7-day post-surgery period are presented in Figure 13.

The cumulative activity counts for the control (surgery only), healthy, mesh/nanofibers, and mesh/nanofibers/drugs groups were 4568 ± 1377, 9637 ± 596, 5815 ± 857, and 7945 ± 1266, respectively. As expected, the animals in the control (surgery only) exhibited significantly inferior activity to healthy animals (*p* < 0.01). Rats that were implanted with drug-eluting mesh showed a higher trigger count than the group with no drugs (non-significant, *p* > 0.05) and a significantly higher number of triggered activities than the control group (*p* < 0.05). Furthermore, they exhibited activity levels similar to the healthy animals (*p* > 0.05). These outcomes indicate that drug-eluting nanofibrous mats are effective in restoring activity levels in the studied animals. This may have contributed to the analgesic effect of ibuprofen which is widely prescribed following oral surgery.

**Figure 14:**
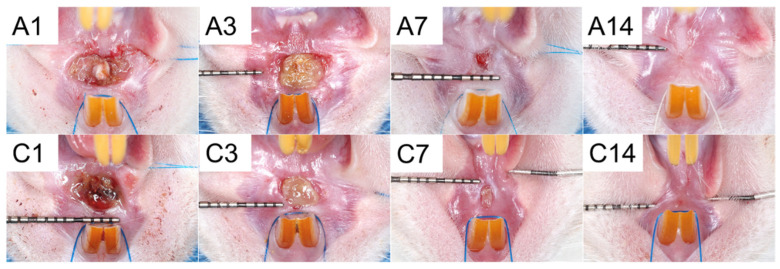
Wound healing in (A) drug-embedded nanofibers with PCL mesh group and (C) control group on days 1, 3, 7, and 14 post-surgery.

Figure 14 shows the healing of wounds at the upper gingiva from Groups A and C on days 1, 3, 7, and 14 after surgery.

Table 1 displays the measurement of the mean area of the wound. These results of clinical wound healing did not show significant differences.

**Figure 15:**
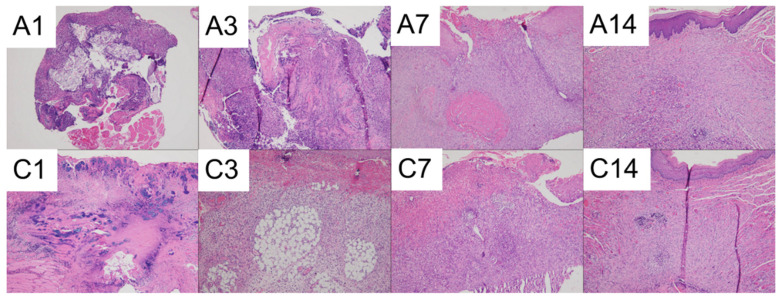
Photomicrographs of (A) Drug-embedded nanofibers with PCL mesh group and (C) Control group on days 1, 3, 7, and 14. (The images were captured using a 10× objective).

The results of the histological analysis are presented in Figure 15.

Both groups displayed significant necrosis along with intense neutrophilic infiltration on days 1 and 3. On day 7, active fibrosis and the formation of granulation tissue were observed underneath the ulcerated surface in both groups. By day 14, the mucosal surfaces had completely healed in both groups. On day 14, foreign body-type giant cells were observed in the drug-embedded nanofibers with the PCL mesh group.

## 4. Discussion

The regeneration or reconstruction of bone to replace oral and maxillofacial defects in tissue engineering requires a porous scaffold. According to reports, nanofibrous membranes offer a distinct topography that enables therapeutic cells to be encapsulated for six weeks, leading to significant cellular infiltration of anti-inflammatory macrophages from the host [32,33]. Successful cell adherence to the scaffold is essential for success, but vastly hydrophobic scaffolds may impede optimal cell colonization. The empirical evidence from this work indicates that incorporating water-soluble pharmaceuticals into nanofibrous membranes improves their hydrophilicity, thus accelerating the necessary tissue proliferation for bone repair.

Kumbar et al. [34] studied nanofibrous membranes produced through electrospinning using scaffolds with different fiber diameters. They discovered that human fibroblasts showed significantly greater growth on electrospun PLGA fiber mats with fiber diameters ranging from 350–1100 nm. The electrospun nanofibers in this work were of optimal size and had a porous structure that allowed drug-eluting nanofibers to generate the biological structural constituents of the extracellular matrix (ECM) for cells to proliferate on [35,36].

This study developed hybrid resorbable 3D-printed mesh/electrospun nanofibrous ibuprofen and EGF-eluting mats for alveolar ridge preservation. Ibuprofen is a nonsteroidal anti-inflammatory drug and blocks the action of cyclooxygenases, which are involved in the production of prostaglandins, to alleviate inflammation, pain, and fever. This mechanism acts peripherally, so ibuprofen can be delivered locally to the site of inflammation or pain [23,37]. In the present study, we demonstrated the effect of analgesics on regaining activities by implanting the drug-eluting meshes into the socket. In addition, the risk of systemic side effects can be minimized through local delivery of pharmaceuticals. EGF is a molecule that plays a role in cell information and can assist with tissue proliferation, angiogenesis, and wound healing [25]. The activation of the mitogen-activated protein kinase (MAPK) pathway and the phospholipase-Cγ (PLCγ) pathway are the primary physiological and biochemical mechanisms and transmission pathways for EGF. These pathways regulate cell proliferation and differentiation, respectively, to promote wound healing [38]. However, the impact of EGF on wound healing remains disputed, and the efficacy of various local delivery methods and concentrations can vary [39,40]. In this study, EGF was embedded into a nanofibrous membrane and applied to the wound. While this approach may cause a foreign body reaction, it did not significantly delay wound healing compared to the control group.

The drug-loaded nanofibers showed inferior fiber dimension to the pristine nanofibers. In a polymer/drug composite, the polymer plays as the component to withstand the external electrical force while performing electrospinning. The addition of the drug reduces the polymer in the composite matrix, making it easier to stretch. Sizes of spun nanofibers diminished accordingly. Additionally, the hollowed core structure of the sheath-core nanofibers possesses less strength to resist the exterior electric force, thus making the fibers prone to be elongated. Spun nanofibers thus have smaller diameter distribution.

Drug elution from a degradable implant containing drugs can be divided into three phases: burst release, diffusion-dominated discharge, and degradation-controlled elution [41,42]. During the spinning process, a significant portion of the loaded pharmaceuticals becomes trapped within the bulk of the PLGA matrices. Nevertheless, some of the drugs may be distributed on the surface of the nanofibers, leading to the preliminary peak release. Following this burst, drug discharge is controlled by both diffusion and polymer degradation. Therefore, a gradual decreasing discharge of ibuprofen was observed. Due to the shielding effect of the sheath PLGA material, no burst release of EGF was found on day 1. Rather, gradual diminishing peaks were noted on days 9, 14, and 20, mainly due to the combined influence of drug diffusion and polymer degradation. Additionally, compared to the in vitro release that displays a burst release, no obvious burst was realized in the in vivo results. The reason for the reduced burst release may be attributed to the generally slower metabolism in vivo compared to in vitro.

Certain limitations were present in the current investigation, which used a rat model to evaluate mats containing drugs and EGF. The extent to which the current findings can be generalized to alveolar ridge preservation in humans remains unclear and warrants further research. We have made plans to pursue this avenue of investigation.

## 5. Conclusions

We utilized 3D printing and electrospinning methods to manufacture resorbable mats that incorporate drugs and biomolecules for alveolar ridge preservation. In vitro testing of the nanofibrous mats revealed a sustained discharge of ibuprofen and EGF for over 4 weeks. In vivo animal testing demonstrated that animals implanted with the hybrid ibuprofen/EGF-eluting mats exhibited significantly greater mobility compared with animals without implants. The histological assay showed no negative effects of the drug/biomolecule-eluting implants. The degradable mats may eventually find use in humans for alveolar ridge preservation.

## Figures and Tables

**Table 1:** The mean area of wound (mm^2^) of Groups A and C.

Group	Day 1	Day 3	Day 7	Day 14
A (Mesh + Drug)	16.07	9.55	4.03	0
C (Control)	12.99	9.59	3.80	0

## Data Availability

All data have been included in the manuscript.
